# Reply to: The G protein preference of orexin receptors is currently an unresolved issue

**DOI:** 10.1038/s41467-023-38765-2

**Published:** 2023-06-01

**Authors:** Jie Yin, Yanyong Kang, Aaron P. McGrath, Karen Chapman, Megan Sjodt, Eiji Kimura, Atsutoshi Okabe, Tatsuki Koike, Yuhei Miyanohana, Yuji Shimizu, Rameshu Rallabandi, Peng Lian, Xiaochen Bai, Mack Flinspach, Jef K. De Brabander, Daniel M. Rosenbaum

**Affiliations:** 1 Department of Biophysics, The University of Texas Southwestern Medical Center, 6001 Forest Park Road, Dallas, TX 75390 USA; 2 Takeda Development Center Americas, Inc., 9625 Towne Centre Drive, San Diego, CA 92121 USA; 3 Takeda Pharmaceutical Company Ltd., 26-1 Muraoka-Higashi, 2-Chome, Fujisawa, Kanagawa 251-8555 Japan; 4 Department of Biochemistry, The University of Texas Southwestern Medical Center, Dallas, TX 75390 USA; 5 BioHPC at the Lyda Hill Department of Bioinformatics, The University of Texas Southwestern Medical Center, Dallas, TX 75390 USA; 6 Present Address: Chinese Institute for Brain Research, No. 26 Science Park Road, Zhongguancun Life Science Park, Changping District, Beijing, China

**Keywords:** G protein-coupled receptors, Cell signalling, Cryoelectron microscopy, Receptor pharmacology

**replying to** J. P. Kukkonen *Nature Communications* 10.1038/s41467-023-38764-3 (2023)

Kukkonen challenges our interpretation of data in Figure 5d of our paper^1^. In this experiment, we co-transfected HEK293 cells with CMV-driven overexpression plasmids for OX_2_R *and* G_αi1_ along with a pGloSensor cAMP reporter construct, and measured diminished cAMP levels with OX_2_R agonists after forskolin stimulation. Results from this experiment showed a weak reduction in cAMP, without a characteristic low-nanomolar EC_50_ for either TAK-925 or Orexin B (in contrast to the parallel inositol phosphate accumulation assay with overexpressed OX_2_R and G_αq_). We acknowledge the validity of Kukkonen’s arguments that the cAMP data could also be explained by a combination of signaling through different G protein subtypes and/or regulation by PDEs. We also appreciate his arguments that the G protein selectivity or promiscuity exhibited by OX_2_R will depend on the cell line and experimental setup, and has not been definitively established in CNS neurons.
